# Findings of pilot study following the implementation of point of care intraoperative PTH assay using whole blood during surgery for primary hyperparathyroidism

**DOI:** 10.3389/fendo.2023.1198894

**Published:** 2023-08-25

**Authors:** Rahul Mohan Kumar, Arslan Pannu, Emily Metcalfe, Mesfin Senbeto, Saba P. Balasubramanian

**Affiliations:** ^1^Sheffield Endocrine Surgery Unit, Sheffield Teaching Hospitals, Sheffield, United Kingdom; ^2^School of Medicine and Biomedical Sciences, University of Sheffield, Sheffield, United Kingdom

**Keywords:** parathyroid hormone (PTH), Intraoperative PTH (IOPTH), parathyroidectomy, NBCL CONNECT, point of care (POC)

## Abstract

**Objective:**

To report findings of pilot study using a novel point of care (POC) intraoperative parathyroid hormone (IOPTH) assay for parathyroid hormone (PTH) using whole blood during surgery for primary hyperparathyroidism (PHPT).

**Methods:**

Patients undergoing surgery for primary hyperparathyroidism from March to November 2022 where intraoperative PTH assay was performed using the NBCL CONNECT IOPTH and the laboratory PTH assay were included (group 1). The biochemistry results were reviewed to determine concordance between NBCL and lab PTH values and diagnostic test parameters of the NBCL CONNECT assay. ‘In-theatre’ times were then compared with a historical cohort (group 2) where the lab-based IOPTH assay alone was used.

**Results:**

Of the 141 paired samples in group I, correlation between NBCL and the lab assay was high (rho=0.82; p<0.001). PTH levels using the NBCL assay dropped satisfactorily (>50% of the basal or 0 min sample; whichever was lower – i.e. positive test) in 23 patients; giving a positive predictive value of 100%. Of the 9 patients that did not demonstrate a drop, two were true negative (negative predictive value of 22%) leading to cure after excision of another gland. Group 1 (150 mins) had a significantly shorter ‘in-theatre’ time compared to group 2 (167 mins) (p=0.007); despite much higher use of near infra-red autofluorescence (NIRAF) (72% vs 11.6% in group I and 2 respectively).

**Conclusion:**

The NBCL CONNECT POC IOPTH assay gives comparable results to lab based PTH assays and can be performed without need for a centrifuge or qualified technicians. Surgeons, however, need to be aware of the potential for false-negative results.

## Introduction

Parathyroidectomy (parathyroid surgery) is the definitive treatment for primary hyperparathyroidism (PHPT) and is curative in around 95% of patients (1). The primary pathology is usually benign and may affect one (single gland disease – SGD) or multiple (multi gland disease – MGD) parathyroid glands; although SGD accounts for ~ 80% of patients (2). The traditional procedure of bilateral neck exploration (BNE) has now largely given way to a targeted or unilateral neck exploration (TP/UNE) in a significant proportion of patients. Currently, TP/UNE accounts for well over 50% of parathyroid surgery in the UK(BAETS) (3). However, this figure is slightly lower (~40-50%) in tertiary centres (2); probably due to the higher proportion of scan negative disease.

In a bilateral neck exploration, dissection to locate/identify all 4 glands has the potential to damage normal glands, increase risk of recurrent laryngeal nerve injury, increase operating time and is reliant on operator experience and judgment to distinguish normal from abnormal glands. This can lead to higher rates of post-surgical hypoparathyroidism (POSH) with significant short-term and long-term morbidity (2). Minimally invasive parathyroidectomy (MIP), in the form of unilateral neck exploration (UNE) or targeted parathyroidectomy (TP) is guided by preoperative identification of an enlarged gland on imaging and appeals to both surgeons and patients given that ~85% of patients have single gland disease (4). This approach may potentially be associated with lower morbidity (5) and reduces hypoparathyroidism to negligible levels. The main limitations of this approach are the potential to miss multi gland disease and increasing failure rate; unless accompanied by frozen section or intra-operative PTH assay (IOPTH) (6).

Failure to cure PHPT is well-recognised (5, 7) potentially due to inability to identify an enlarged gland(s) in eutopic or ectopic locations, failure to recognise presence or extent of multi gland disease (7). Prediction of multi gland disease using defined criteria have been proposed as a solution (8–10). However, published evidence from this unit has shown that these criteria based on pre- and/or intra-operative features do not accurately differentiate single from multi gland disease (2).

Intra-operative parathyroid hormone monitoring (IOPTH) may improve success rates and reduce need for extensive dissection (11, 12). The failure of PTH to drop after excision of a single large parathyroid is a clear indicator of multi gland disease and may reduce failure rates of MIP (5). IOPTH may also be an alternative to frozen section confirmation of parathyroid tissue. Minimally invasive parathyroidectomy utilising IOPTH has been shown to improve cure rates and reduce morbidity in some (13), but not all studies (14–16). Potential limitations include increase in cost and the variably reported risk of false positive and false negative results, which may in turn be due to variations in half-life of PTH (17), definition of baseline value (18) and timing of post-excision assessment (19). The UK National Institute for Health and Care Excellence (NICE) guidance on surgery for PHPT in 2019 (20) did not support routine use of IOPTH given its cost, despite noting a ‘marginal benefit’. However, several UK centres use IOPTH and a recent study has demonstrated cost savings (20), challenging the current NICE guidance. In a recent meta-analysis that demonstrated MIP to be associated with similar success but lower complication rates and operating time compared to BNE (21); however, most (84%) of studies in this review used IOPTH. The shorter operating time in the MIP arm (facilitated by IOPTH) has also enabled the adoption of parathyroidectomy as a day case procedure (13).

The intact PTH hormone has a very short half-life (2-4 minutes) while C-terminal fragments (which account for 80% of total circulating PTH have a much longer half-life (22). There are three generations of PTH assays (23), but currently, most labs use the second-generation assay.

Several ‘lab based’ and ‘point of care’ systems are now available to enable intraoperative assessment of PTH levels. All these assays typically require blood to be collected in a EDTA vial, centrifuged and the serum used to assay PTH. The reported time for the results to be available ranges from 8-16 minutes (24).

Numerous guidelines help define an ‘adequate’ PTH fall that guide the decision to terminate the operation; thereby avoiding the need for further dissection (25). The guidance varies in how to define baseline values, the number and timing of measurements and the definition of an adequate fall in PTH (25).

The standard practice in Sheffield Teaching Hospitals was to obtain a baseline (after induction, but prior to incision) measurement, a further measurement just prior to gland excision and a sample 15 minutes after excision. These three blood samples were sent together to the central lab for PTH assessments. A ‘positive’ result would be a >50% fall of PTH in the 15-minute sample compared to the pre-incision or pre-excision value. This was left to the discretion of the surgeon and depended on consideration of imaging, patient, and disease related factors.

The Novel Biomarkers Catalyst Lab (NBCL) CONNECT analyser enables an intraoperative assessment of PTH levels available withing a few minutes using an intact PTH (second-generation) assay in a single step (sandwich immunoassay), performed on whole blood. This pilot study aims to evaluate the results of surgery for primary hyperparathyroidism following the implementation of the NCBL CONNECT assay in one centre. The objectives of this study include:

1. Determine the accuracy of the results of the NBCL CONNECT assay by comparison with the lab ‘IOPTH’ assay and postoperative adjusted calcium and PTH levels.
2. Compare ‘in-theatre’ time in groups of patients operated using the lab ‘IOPTH’ assay and the ‘NBCL CONNECT’ assay.
3. Explore the ‘false positive’ and ‘false negative’ results obtained by the NBCL CONNECT assay.

## Methods

Patients with primary hyperparathyroidism operated using the NBCL CONNECT assay in one UK centre were initially considered for inclusion in the study (Group I). These patients were later compared to a historical cohort (Group II) where the lab IOPTH assay was used. The duration of recruitment was from March 2022 to November 2022 for the NBCL cohort. The pre-operative work up and surgery was performed in a manner consistent with local practice. The operations were performed by or under the supervision of a single consultant surgeon. For all included patients, after intubation and positioning, an ultrasound scan was usually performed to confirm the findings of preoperative imaging and determine the relationship of any enlarged parathyroid glands to the thyroid, carotid artery and internal jugular vein. Following this a bilateral superficial plexus nerve block was performed under ultrasound guidance.

During surgery, samples of venous blood were taken and tested for PTH using both the NBCL CONNECT assay and standard laboratory (Roche) assay simultaneously. The NBCL CONNECT assay was done immediately and used to guide the extent of surgery; while the lab assay was performed later and did not guide surgical decision making. Both these assays are 2nd generation PTH assays.

In general, the timing of blood tests was baseline or pre-incision (after intubation prior to skin incision), time zero or pre-excision (at identification of the enlarged parathyroid gland), 5 minutes and 10 minutes after devascularization and/or excision or the enlarged parathyroid gland. However, in some instances, the timings varied based on the operative findings and the judgement of the operating surgeon.

A positive test result on the NBCL CONNECT assay was defined as a drop in PTH of >50% of the lower of the baseline or ‘0-minute PTH’. A negative test result (unsatisfactory drop in PTH) was defined as a drop in the PTH <50% of the higher of the baseline or ‘0-minute PTH’. If the drop in 10-minute PTH was >50% of the higher of the two pre-excision values but <50% of the lower of the two pre-excision values, the test was considered equivocal.

A positive test result warranted termination of neck exploration. A negative result warranted further neck exploration and search for enlarged parathyroid glands. In the equivocal test results the decision to terminate the operation was made by the operating surgeon based on the clinical circumstances. All these decisions were based on the results of the NCBL assay.

After surgery, postoperative care depended on whether patients were managed as ‘day case’ or whether they were admitted for overnight stay. Patients who were able to go home the same day in accordance with a local ‘day case’ protocol went home on calcium and vitamin D supplements and had a telephone appointment the day after surgery. Patients managed as ‘inpatients’ had serum calcium and PTH measurements the day after surgery and were sent home with supplements dependent on the results of these blood tests.

The primary endpoints were cure rates and correlation between the PTH levels assessed by the NBCL CONNECT and the laboratory assays. Cure was defined as normalisation of PTH and adjusted calcium in postoperative blood samples. Secondary endpoints included assessment of true-positive and true-negative results of the NBCL assay; the gold standard being the laboratory values. Additional endpoints included in-theatre times for patients which was recorded from the operating theatre database where-in the times are logged in by staff in the theatre room. These times were compared to times in another historical cohort of patients in where the laboratory PTH assay was used to guide surgical decision making. Due to the short-term nature of the study, data on longer term follow up was not available.

Demographics, perioperative biochemistry and surgical details were collected, and data was anonymised before analysis. Descriptive analyses included frequencies and percentages for qualitative data, mean and standard deviation for quantitative data that was normally distributed and median and range for quantitative data that was not normally distributed. Inferential methods include the use of Pearson rho correlation coefficient for the correlation of the NBCL CONNECT and the lab PTH assays. Diagnostic parameters of true and false positive and negative tests were calculated for the NBCL CONNECT in comparison to the lab PTH assays. Comparisons of in-theatre times between the two groups were done using the Mann-Whitney U test.

The NBCL CONNECT assay is currently approved for clinical use in the UK and is being introduced in several UK centres. This project was undertaken as a service evaluation project in one UK centres. For this reason, it was not deemed necessary to apply for ethics approval. Permission was however obtained from the directorate of general surgery in Sheffield Teaching Hospitals. No patient identifiable data was accessed by anyone outside the clinical teams. For this reason, informed patient consent was not deemed necessary.

## Results

In Group I, 32 patients where the NBCL assay was used during surgery for primary hyperparathyroidism were included during the period March 2022 to November 2022. These included 26 females and 6 males. The median (interquartile range) age of the included patients was 60 (48-72) years. MIBI and USS were performed on all patients. Majority of patients had imaging lateralizing to one side (84% on ultrasound and 75% on MIBI). Concordance between MIBI scan and pre-op USS was 75.0% (24/32). Of these 24 patients, localization was found to be incorrect in one patient.

This study included 141 paired samples from 32 patients for comparative analysis. Of these, correlation between NBCL and the lab assay was high (rho=0.82; p<0.001). PTH levels using the NBCL assay dropped satisfactorily (>50% of the basal or 0 min sample; whichever was lower – i.e. positive test) in 23 patients; giving a positive predictive value of 100% as seen in **Table 1**. Of the 9 patients that did not demonstrate a drop, two were true negative (negative predictive value of 22%) leading to cure after excision of a further gland. Of the 7 patients with false negative results, further exploration was performed in 5 of them. Sensitivity of the NBCL assay was 77% (95%CI of 58-90%), while specificity was 100% (95%CI of 16-100%). The decision to proceed with further exploration was based on intraoperative findings (including the finding of a second normal gland on that side) and surgical judgement (of the likelihood of single versus multi-gland disease), in combination with the intraoperative PTH results. Biochemical cure with normalization of serum calcium and PTH was achieved in all patients using the NBCL CONNECT assay.

**Table 1:** Confusion matrix of Group I comparing NBCL assay cohort against the lab assay results.

		**Lab assay**	
		**Positive**	**Negative**
**NBCL assay**	**Positive**	True Positive = 23	False Positive = 0
	**Negative**	False Negative = 7	True Negative = 2

PPV=100% (95% CI of 85-100%); NPV=22% (95%CI of 3-60%); Sensitivity=77% (95%CI of 58-90%); Specificity=100% (95%CI of 16-100%).

The patients in Group I (n=32) where the NBCL assay was used from March 2022 to November 2022 were compared with a historical cohort of 47 patients prior to the availability of the NBCL assay (group 2) where the lab assay was used. Patients from group 2 underwent their respective procedures from April 2019 to 2021. The baseline characteristics of the two groups of patients are shown in **Table 2**. Statistically different demographics between both cohorts were noted in the concordance of dual imaging and the use of autofluorescence; both of which were higher in group one. In-theatre times were significantly shorter (p=0.007) in Group I compared to Group II as shown in **Table 2**.

**Table 2:** Comparison of demographics, radiology findings and clinical outcomes in the NBCL assay cohort (Group I) and the lab assay cohort (Group II).

Characteristics			Group I (NBCL assay) (n=32)	Group II (Lab IOPTH assay) (n=47)	P value
Gender, n (%)					
Male			6 (18.7)	10 (20.3)	1.0
Female			26 (81.3)	37 (78.7)	
**Median age (IQR)**			60 (47.50-71.75)	60 (44.0-72.0)	0.90
USS lateralising, n (%)					
Yes			27 (84.4)	33 (70.2)	0.24
No			5 (15.6)	14 (29.8)	
MIBI lateralising, n (%)					
Yes			24 (75.0)	26 (55.3)	0.12
No			8 (25.0)	21 (44.7)	
Concordance of imaging, n (%)					
Yes			24 (75.0)	17 (36.2)	0.002
No			8 (25.0)	30 (63.8)	
Autofluorescence					
AF			23 (72.9)	8 (17.0)	<0.001
No AF			9 (28.1)	39 (83.0)	
**Cure rate n (%)**			32 (100.0)	46 (98.0)	1.0
**In-theatre time (mins)**		Median	150.0	167.0	0.007
		IQR Range	130.25-167.50	151.0-177.0	

## Discussion

Intra-operative parathyroid hormone (IOPTH) assay is an intraoperative tool which aids in detecting a significant fall in PTH levels during surgery predicting cure of primary hyperparathyroidism, while reducing the need for extensive four-gland dissection and associated post-operative complications (26, 27). Arguments against IOPTH are based on the costs of routine implementation, risks of false positive and false negative test results and uncertain effectiveness in improving patient outcomes (28). However, this view has been challenged, with evidence showing that IOPTH may be cost effective by increasing cure rates and reduce operating times; by reducing the costs of investigations and monitoring in patients with failed operations, complications relating to extensive dissection and prolonged operating times (20).

Results from this pilot study using the NBCL CONNECT assay has shown a high correlation with lab assay in 141 paired samples. In addition, no false positive results have been observed in this small cohort. This positive correlation and performance of the assay in predicting biochemical cure has recently been demonstrated in another study (29); which has not yet been published.

However, within the cohort of 32 patients, there were 7 instances of false negative results. It is expected that ongoing improvements in the technology and software will result in reduction in these false negative results.

When comparing theatre times between the NBCL CONNECT and lab assays, a significant reduction in ‘in-theatre’ times was demonstrated in Group I. As previously highlighted, the NBCL connect analyser doesn’t require centrifugation, can be used by theatre staff and is able to provide results using whole blood within minutes. Conversely, the lab assay would require transport to the laboratory and subsequent analysis by a trained technician, this process is eliminated when using the NBCL CONNECT and may explain the reduction in comparative theatre times.

However, it must be highlighted that in the period in which patients from group 2 underwent their operations, IOPTH was not routinely used for all cases and specifically selected for cases deemed to be challenging by the surgeon. It could be argued that this is a more difficult patient cohort, therefore, prolonging theatre time. A counterpoint to this would be the increased use of auto-fluorescence in group 1 (72.9%) vs group 2 (17.0%) which also would increase ‘theatre-time’.

## Limitations

This study has a small sample size, is observational in nature and has a control arm that is historical. Given the retrospective nature of this study, elements of selection bias such as the potential use of IOPTH in ‘more difficult cases’ in the control arm were unavoidable. In addition, the increased use of NIRAF in the NBCL CONNECT arm could potentially confound the outcomes. This study has not compared the use of NBCL assay with currently available ‘in-theatre’ assays; as these are currently not available. Despite this, the results of this pilot study are important and highlight the potential utility of this technology and the concern about false negative results.

## Conclusion

The NBCL CONNECT assay showed high correlation with compared with lab based PTH assays. Its advantages over other assays lie in its potential to reduce theatre time and shorter turnaround times. However, surgeons must be aware of the potential of false negatives results, consider intraoperative findings and assess the potential for multi-gland disease; prior to making decisions on further exploration. Further work is ongoing to reduce the risk of false negative results.

## Data availability statement

The original contributions presented in the study are included in the article/supplementary material. Further inquiries can be directed to the corresponding author.

## Author contributions

RM: substantial contributions to the conception or design of the work; or the acquisition, analysis, or interpretation of data for the work. Writing and submission of final manuscript. AP: substantial contributions to the conception or design of the work; or the acquisition, analysis, or interpretation of data for the work. EM: substantial contributions to the conception or design of the work; or the acquisition, analysis, or interpretation of data for the work. MS: substantial contributions to the conception or design of the work; or the acquisition, analysis, or interpretation of data for the work. SB: substantial contributions to the conception or design of the work; or the acquisition, analysis, or interpretation of data for the work. Writing and submission of final manuscript.
